# Towards multiple readout application of plasmonic arrays

**DOI:** 10.3762/bjnano.2.54

**Published:** 2011-08-30

**Authors:** Dana Cialla, Karina Weber, René Böhme, Uwe Hübner, Henrik Schneidewind, Matthias Zeisberger, Roland Mattheis, Robert Möller, Jürgen Popp

**Affiliations:** 1 Institute of Physical Chemistry and Abbe Center of Photonics, Friedrich-Schiller-University Jena, Helmholtzweg 4, 07743 Jena, Germany; 2 Institute of Photonic Technology, Albert-Einstein-Straße 9, 07745 Jena, Germany

**Keywords:** fluorescence, multiple readout, plasmonic array, surface-enhanced fluorescence (SEF), surface-enhanced Raman spectroscopy (SERS)

## Abstract

In order to combine the advantages of fluorescence and surface-enhanced Raman spectroscopy (SERS) on the same chip platform, a nanostructured gold surface with a unique design, allowing both the sensitive detection of fluorescence light together with the specific Raman fingerprint of the fluorescent molecules, was established. This task requires the fabrication of plasmonic arrays that permit the binding of molecules of interest at different distances from the metallic surface. The most efficient SERS enhancement is achieved for molecules directly adsorbed on the metallic surface due to the strong field enhancement, but where, however, the fluorescence is quenched most efficiently. Furthermore, the fluorescence can be enhanced efficiently by careful adjustment of the optical behavior of the plasmonic arrays. In this article, the simultaneous application of SERS and fluorescence, through the use of various gold nanostructured arrays, is demonstrated by the realization of a DNA detection scheme. The results shown open the way to more flexible use of plasmonic arrays in bioanalytics.

## Introduction

Fluorescence spectroscopy and microscopy is one of the most important analytical techniques in the life sciences and medicine. Due to its extreme sensitivity, fluorescence allows investigations on a single-molecule level [1]. Fluorescence in bioanalytics is mostly used for tracking intrinsic fluorophors (autofluorescence) or special fluorescence labels, which selectively bind to special cellular functional groups, such as proteins or nucleic acids [2–3]. However, the broad emission spectrum of the fluorescence reporter molecules prevents the parallel detection of several fluorescent dye labels by fluorescence microscopy.

Raman spectroscopy, i.e., the excitation of vibrational modes through inelastic light scattering, allows one to obtain highly specific molecular fingerprint information without the need for external labels. The drawback of the intrinsically small Raman scattering cross sections not allowing for trace analytics and fast detections times can be overcome by applying surface-enhanced Raman spectroscopy (SERS). The enhancement of the inherently small Raman cross sections by applying SERS is based on the strong plasmonic field enhancement at rough metallic surfaces. Since SERS combines the unique fingerprint specificity of Raman with trace level sensitivity, it is a very active topic in (bio)analytics [4–13].

In order to exploit the advantages of both fluorescence and SERS spectroscopy in a single sensor platform, the goal of the work presented here is the development and application of an innovative nanostructured surface that will allow both detection schemes. Thus, several requirements must be fulfilled by the plasmonic array:

(1) Periodically patterned arrays with homogenous signal enhancement must be prepared, because difficulties in the fabrication of metallic surfaces with reproducible signal enhancement hamper the routine application of SERS as a (bio)analytical tool [14]. (2) A patterned plasmonic active film is essential for detecting both fluorescence and SERS signals through a single plasmonic array, since the optimum distance between the (fluorescent) molecules and the metallic surface, for gaining the maximum signal intensity, is different in the two cases. The most efficient SERS enhancement is achieved for molecules within the first layer of the metallic surface, where the fluorescence signal will be quenched most efficiently. Thus, a parallel detection of fluorescence and SERS is prevented when applying a continuously nanostructured metallic layer, such as roughened metal electrodes, as a sensor array. (3) A further requirement which has to be fulfilled is the realization of large-scale production capacity for applications in (bio)analytics.

One of the most common type of periodically patterned plasmonic arrays is based on the formation of a polystyrene or silicon dioxide bead mask during the production process, such as in nanosphere lithography (NSL) [15–16], film over nanospheres (FON) [17–18], and sculpted SERS substrates [19]. Here, the arrays are tunable by varying the size of the monodisperse polystyrene or silicon dioxide beads. Unfortunately, frequently occurring constructional defects within the mask are transferred to the nanostructured metallic surface. Furthermore, electron beam lithography (EBL) [20] is a promising production technique for periodically patterned plasmonic arrays. We have recently shown that gold nanorhomb arrays produced by EBL [21] provide a homogenous signal enhancement across a large area [22]. By virtue of the patterning process, the optical parameters are tunable by varying both the size of the nanoparticles and the period of the array [23], which leads to design and fabrication strategies of SERS arrays developed to gain a maximum SERS enhancement [24].

Furthermore, the use of our gold nanorhomb arrays fabricated on a quartz wafer allows the binding of analyte molecules also through direct attachment to the quartz surface. Thus, molecule–surface distances from zero to several tens of nanometers (depending on interparticle distances) can be obtained, hence allowing both fluorescence and SERS detection. Finally, once optimized, plasmonic arrays produced by electron beam lithography can also be prepared through nanoimprint techniques, an inexpensive method to manufacture large quantities. Therefore, we report here on the application of such a nanorhomb array on a quartz wafer biochip platform for DNA detection by fluorescence and SERS readout. By doing so, fluorescence microscopy allows for a fast detection of any positive or negative binding event within several seconds. Moreover SERS provides detailed molecular fingerprint information of fluorescence reporter molecules. This work contributes to the development of the more flexible usage of different optical detection schemes on the same chip surface.

## Results and Discussion

**Figure 1:**
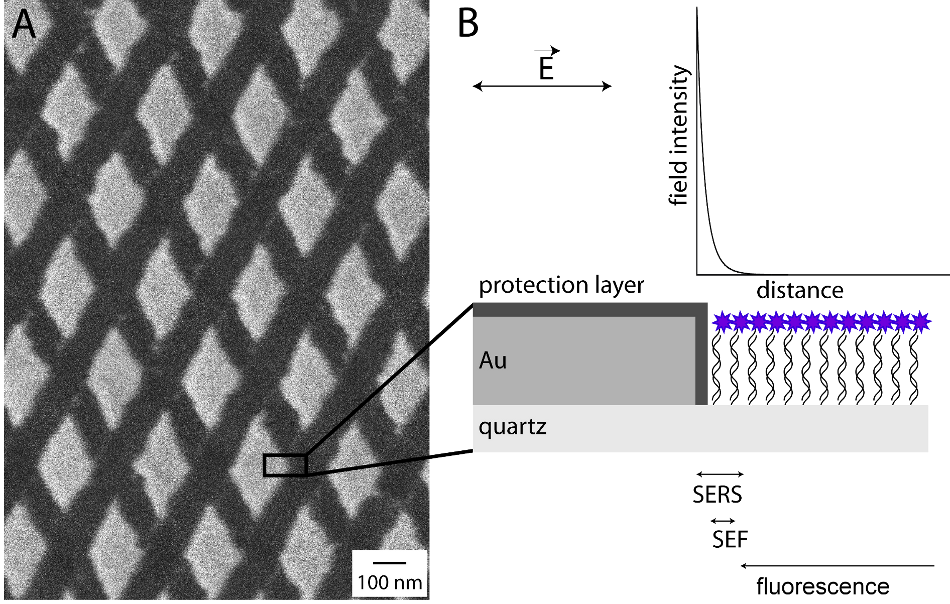
Schematic illustration of gold nanorhombs as plasmonic substrate. (A) The SEM image shows the periodically patterned gold surface. (B) Due to the interaction of the incident light with a metallic nanoparticle, surface plasmons are generated on the metal dielectric interface yielding a strong electromagnetic field with an evanescent decay on the nanoparticle surface. The strong electromagnetic field enhancement is employed for the effective enhancement of the Raman cross section (SERS) and the fluorescence (SEF), as indicating by the arrows.

The work presented in the following reports on the design and application of biosensors based on periodically patterned plasmonic arrays, which can be read out by both fluorescence and Raman spectroscopy, thus utilizing the unique sensitivity of fluorescence spectroscopy and the molecular selectivity of Raman spectroscopy. Therefore, chip surfaces with gold as the plasmonic material and with clearly defined arrays in the range of 200 × 200 µm^2^ were fabricated. The number and position of the plasmonic arrays across the entire sensor chip can be adjusted to the particular research and application of interest. In Figure 1A, the SEM image of a section of the periodically patterned plasmonic array is depicted. Rhombic structures with interparticle distances of ~100 nm were fabricated on a quartz wafer. The size of the single rhomb-shaped gold nanoparticles of the plasmonic arrays used in this study was 50–240 nm along the short axis and 100–750 nm along the long axis. The shape and size of the nanoparticles of the plasmonic array define its optical properties.

In order to illustrate potential applications of these plasmonic arrays for bioanalytics, the arrays were tested for DNA detection. Therefore, capture oligonucleotides were immobilized on the chemically modified quartz surface between the gold nanorhombs. The nonspecific interaction of the DNA strands with the gold surface, through the nitrogen atoms of the DNA bases, was prevented by the formation of an octanethiol SAM as a lipophilic protection layer. Dye-labeled target DNA was incubated on the chip surface and bound efficiently to the complementary capture sequences during the hybridization process. Figure 1B highlights the different readout methods, utilizing the properties of the periodically patterned plasmonic arrays. The excitation of localized surface plasmon resonances (LSPR) at the metal dielectric interface induces a strong electromagnetic field with evanescent decay on the metal surface. This strong field enhancement by the evanescent field can be employed for an effective enhancement of the weak Raman cross section (surface-enhanced Raman spectroscopy – SERS) [25] and also of the fluorescence signal (surface-enhanced fluorescence – SEF) [26]. However, the signal enhancement in SERS and SEF is characterized by different dependencies on the distance between the analyte and metal surface. In order to establish rules for an analyte–metal-surface, distance dependent, signal enhancement, scanning probe microscopy (SPM)-based measurements in combination with an optical readout were performed by several research groups: Roth et al. applied distance dependent tip-enhanced Raman spectroscopic (TERS) measurements, where SERS is combined with the SPM technique AFM (atomic force microscopy). These distance dependent TERS studies revealed that the highest signal intensities can be found for the smallest distance between tip and surface and moreover that the enhancement decays on a length scale of approximately 10 nm [27]. Furthermore, Anger et al. investigated the distance dependent enhancement of single-molecule fluorescence. The most efficient fluorescence enhancement was detected for distances in the range of 3–7 nm, whereas for shorter distances the molecular fluorescence was quenched [28]. Since the plasmonic behavior of a SPM probe for tip-enhanced near-field optical microscopy is comparable with that of a single metallic nanoparticle, the usage of periodically ordered plasmonic arrays should allow the application of SERS readout for molecule–metal surface distances up to 10 nm and SEF for analyte–metal distances of around 5 nm (Figure 1B). For distances of more than 20 nm the molecule remains more or less unaffected by the strong electromagnetic field enhancement, so normal fluorescence should be detectable.

**Figure 2:**
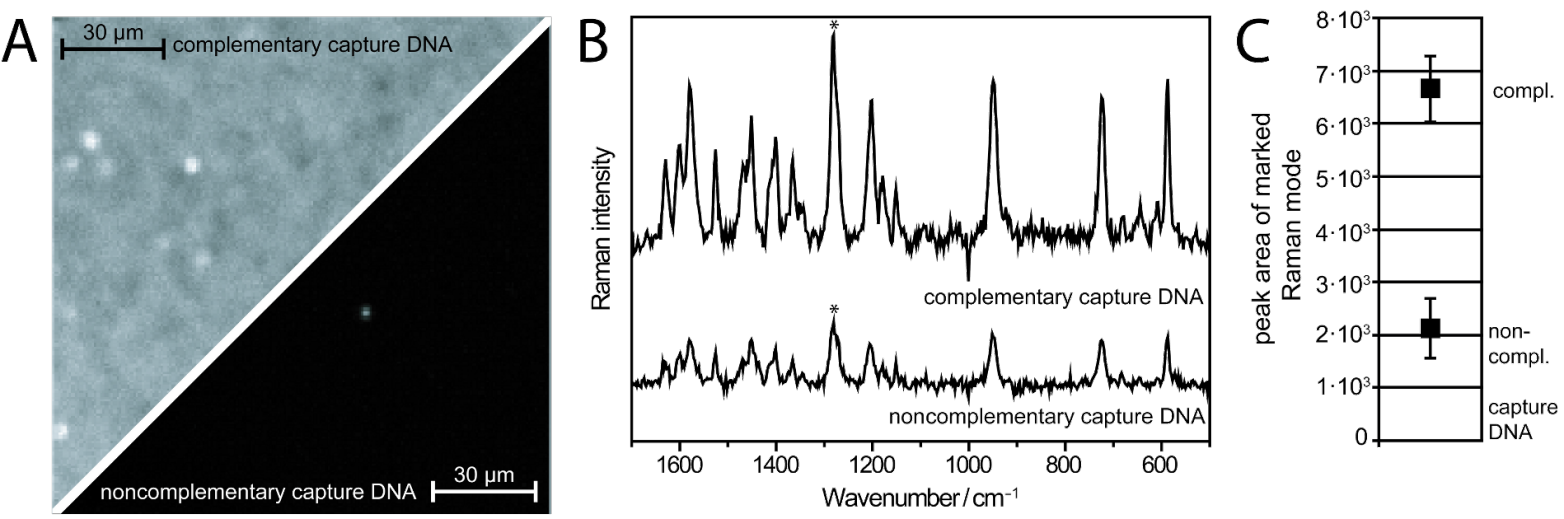
Application of the same plasmonic array for fluorescence and SERS readout. (A) Here, the fluorescence signal is shown in the upper-left and lower-right, for the complementary and noncomplementary capture and dye-labeled target DNA sequences, respectively. A bright fluorescence signal was detected for the complementary case due to the effective formation of the DNA double helix. (B) Raman signature (background-corrected mean value spectrum) of the Cy3.5 label, which is attached to the target DNA. (C) As more dye-labeled DNA molecules are attached to the surface in the complementary case, due to the double helix formation, the Raman signal is more intense.

In order to test the simultaneous application of fluorescence readout and SERS measurements on one common biochip platform, a DNA detection scheme based on the usage of a well-known fluorescence dye label (cyanine dye Cy3.5) was performed. In doing so, complementary and noncomplementary (here: negative control) capture DNA was immobilized on the sensor surface. Furthermore, the biochip was treated with dye-labeled target DNA, which binds to its complementary sequence. In a first test, fluorescence spectroscopy as one of the standard methods in bioanalytics was performed for a fast and easy control of the biochemical binding process: In the complementary case a bright fluorescence signal was detected, whereas under the same conditions no fluorescence signal was found for the noncomplementary case (Figure 2A). In order to gain more detailed information, SERS was employed as a readout technique for the same biosensor. The detected mean value SERS spectra, shown in Figure 2B, are dominated by contributions of the Cy3.5 label (see therefore the reference spectrum in [29]). The detected background signal, which can be attributed among others factors to the fluorescence emission of the dye, was subtracted from all SERS spectra for better visibility. An intense SERS signal of the reporter molecule Cy3.5 was detected due to the double helix formation in the complementary case. By insertion of mismatches within the DNA strand, the binding efficiency of the dye labeled target DNA and moreover the SERS signal was decreased for the noncomplementary case (Figure 2C). Thus, the established biosensor allowed the distinction between a complementary and a noncomplementary binding of target DNA through both a fluorescence and SERS detection scheme. This will be useful for the flexible usage of the same biochip platform in applying different detection schemes.

**Figure 3:**
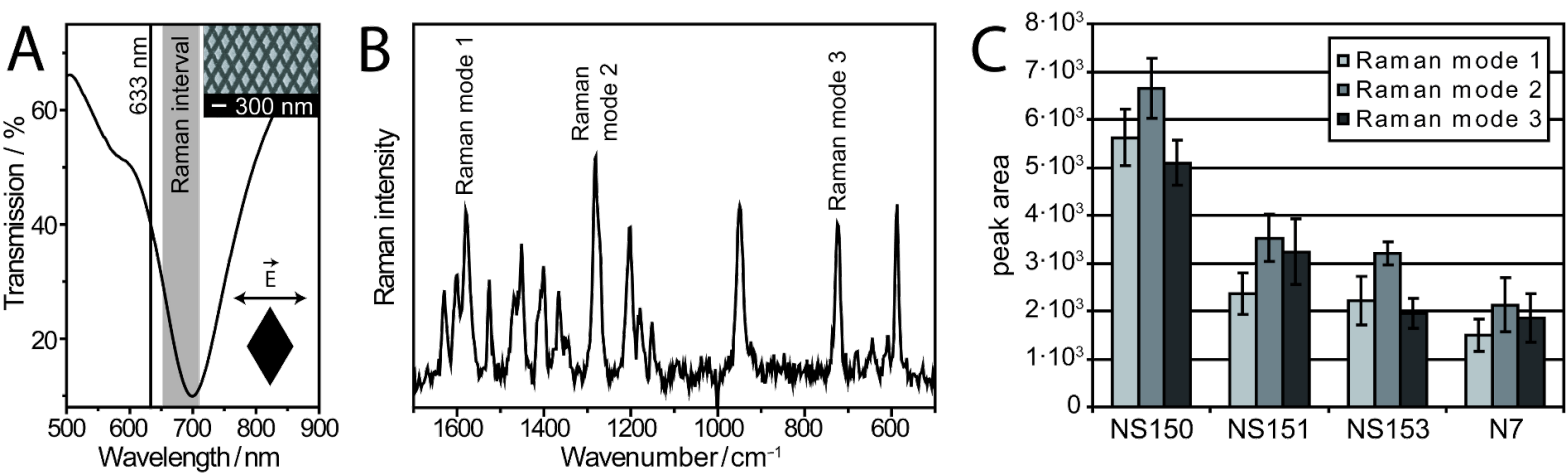
Mismatch-selective SERS investigation. (A) Transmission minimum at around 700 nm indicates the surface plasmon excitation along the short rhomb axis (inset: SEM image of periodically distributed nanorhombs). The excitation wavelength of 633 nm and the Raman interval matches the plasmonic profile. (B) The background-corrected Raman spectrum is dominated by contributions from the dye-label Cy3.5. The target DNA sequence is complementary to that of the capture DNA. (C) In order to illustrate the sequence-specific Raman intensity of three different Raman bands (marked in Fig. B), capture DNA with the complementary sequence (NS150), one mismatch (NS151), three mismatches (NS153), and the non-complementary sequence (N7) with respect to the Cy3.5-labeled target DNA were immobilized on the biochip surface. The strongest Raman signal intensity was detected for the complementary case, which indicates the efficient double helix formation.

In order to achieve a maximum SERS enhancement, the excitation wavelength should lie within the plasmonic absorption. As shown in Figure 3A the optical transmission minimum at around 700 nm indicates the surface plasmon excitation along the short rhomb axis. Thus the Raman excitation wavelength of 633 nm is in resonance with the short wavelength tail of the plasmon band. However, the emitted Stokes Raman-scattered light of modes that are also in resonance with the surface plasmon will also be enhanced due to the plasmon resonance of the nanoantenna. This effect is described as the second part of the electromagnetic SERS mechanism [30–31]. Recently we [24] investigated this contribution of secondary emission enhancement to the overall SERS signal, utilizing the anisotropic character of gold nanorhomb arrays. Since the signal enhancement follows the plasmonic profile, fabrication strategies were developed for the vis and NIR spectral region. Here, the geometry of the gold nanorhombs was optimized by numerical calculations to efficiently improve the emission enhancement process. In Figure 3B a typical SERS spectrum is depicted for the complementary case concerning the capture and dye-labeled target DNA. The background-corrected fingerprint signature is dominated by contributions from the dye-label Cy3.5. In order to investigate the mismatch specificity of the binding process on the biochip surface, capture DNA was immobilized on the free quartz surface with the complementary sequence (NS150), one mismatch (NS151), three mismatches (NS153), and the noncomplementary sequence (N7), with respect to the Cy3.5-labeled target DNA. Figure 3C shows the Raman signal intensity of three prominent Raman modes, as determined by their peak areas. The mean values of the signal intensities are plotted for each of the tested capture DNA sequences. The largest signal intensities were found for the complementary case (NS150) due to the most specific interaction between the capture and target molecule. The Raman signal intensities for the mismatch (NS151, NS153) cases are lower than for the complementary (NS150) case, thus demonstrating the mismatch specificity of the used protocol. In addition, the signal intensity for the negative control (N7) is significantly lower than for the complementary case. Thus the established biochip is an appropriate tool for sequence specific SERS investigation and application to DNA analytics.

**Figure 4:**
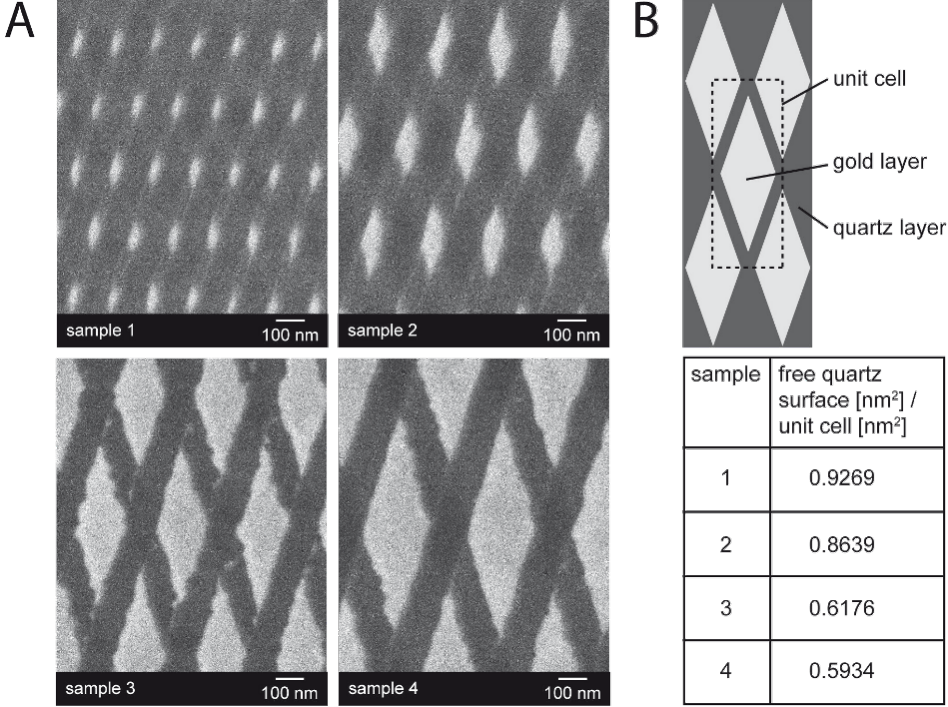
Various plasmonic arrays, which were used for a fluorescence comparison study. (A) SEM images of the used plasmonic arrays illustrate the nanoparticle size and the interparticle distance, which is in the range of 100 nm. (B) Estimation of the free quartz surface area per unit cell.

Due to the strong electromagnetic field enhancement, the fluorescence intensity of chromophores can also be enhanced in close vicinity to the metallic nanoparticles (SEF) [32–33]. In order to verify the amount of fluorescence signal enhancement that is due to coupling with a strong electromagnetic field, various plasmonic arrays were used for this study. The SEM images are depicted in Figure 4A. As mentioned above, the plasmonic samples were arrays of gold nanorhombs mounted on a quartz wafer, with interparticle distances in the range of 100 nm. The fluorescent dye was bound to DNA strands on the free quartz surface. Binding to the gold layer was hindered by a lipophilic protection layer. Thus the detected fluorescence intensity was strongly correlated with the area of free quartz surface per unit cell (Figure 4B). The lower the density of gold per unit cell, the higher the fluorescence intensity should be. Furthermore, the fluorescence enhancement is locally confined to nanosized areas with strong electromagnetic field enhancement, which correspond to the edges of the short rhomb axis when using visible excitation wavelengths [22]. The perimeter of the nanorhomb plays no prominent role in the electromagnetic field enhancement. Due to the large distances between the gold nanoparticles, the fluorescence intensity of molecules bound on the quartz surface remains mainly unaffected by the excitation of the LSPR. Therefore, the detected fluorescence intensity was normalized with respect to the free quartz surface area per unit cell of the various plasmonic arrays.

**Figure 5:**
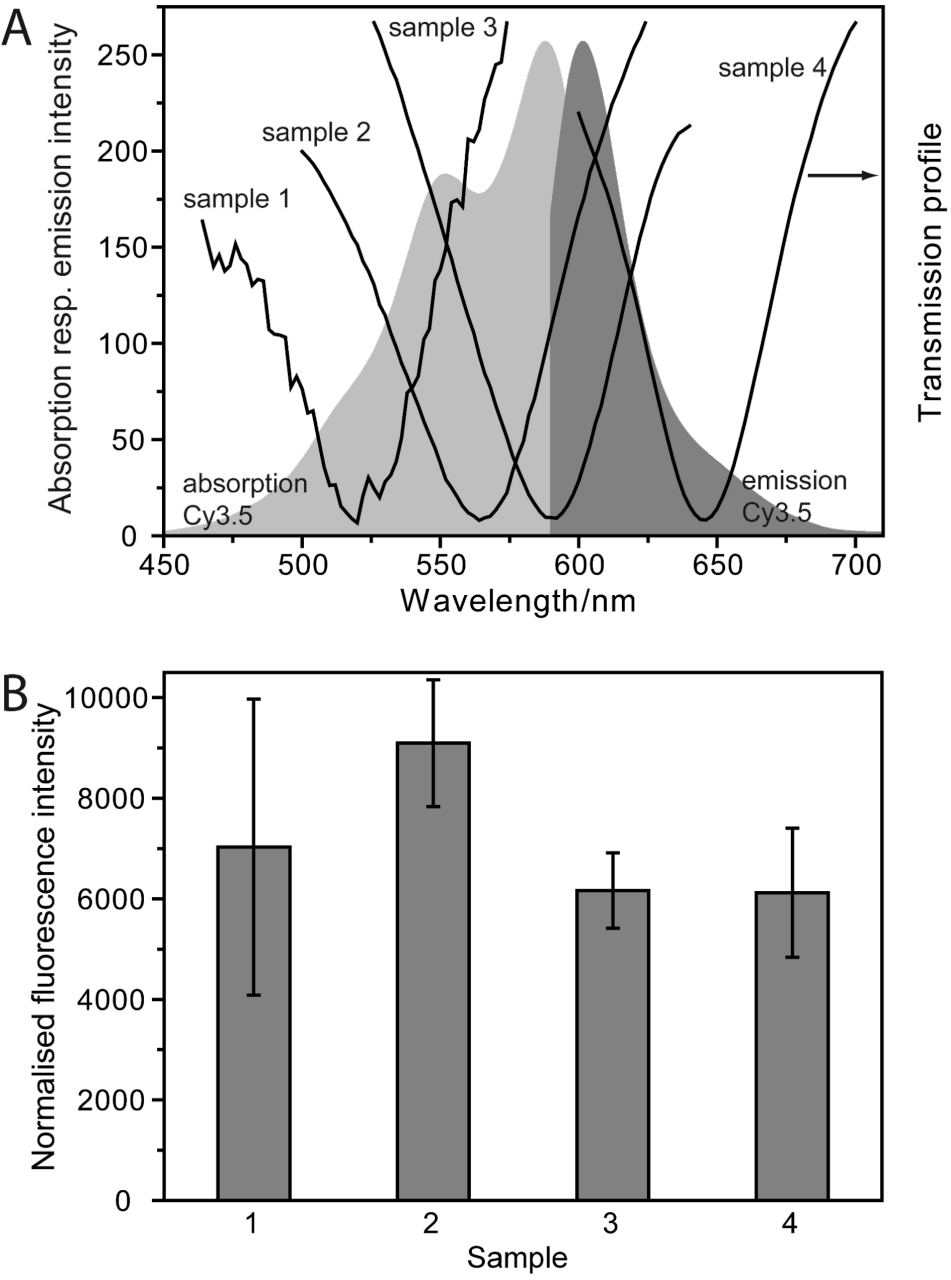
Observation of enhanced fluorescence intensity. (A) Absorption and emission spectrum of the dye-label Cy3.5, attached to a DNA strand, in comparison to the transmission spectra of various plasmonic arrays. The transmission minima (indicating the LSPR excitation) overlap with the absorption and emission spectra differently. (B) The detected fluorescence intensity is normalized with respect to the free quartz surface area per unit cell. The highest signal intensity was detected for sample 2, which may be attributed to a fluorescence enhancement mechanism.

Capture DNA was immobilized on the free quartz wafer. The dye-labeled target DNA bound to its complementary capture sequence. Thus, the formation of the DNA double helix was indicated by the fluorescence signal of the Cy3.5 label. The absorption and emission spectra of the fluorophore Cy3.5 and moreover the plasmonic profiles of the used gold nanorhomb arrays are depicted in Figure 5A for comparison. Since the various plasmonic arrays are characterized by a different size of the individual gold nanorhombs (Figure 4A), the transmission minimum, indicating the excitation of the LSPR, shifts to higher wavelengths with increasing size of the gold nanorhombs. Thus the LSPR peak overlaps with the absorption and emission spectra differently, which may have an influence on the fluorescence intensity. The normalized fluorescence intensity (detected fluorescence/free quartz surface area per unit cell, illustrated in Figure 5B) of sample 2 is surprisingly higher than that for the other plasmonic samples, whereas sample 3 and 4 exhibit comparable signal intensities. The observed fluorescence enhancement with sample 2 opens the way towards systematic SEF investigations with tunable plasmonic arrays. Furthermore, these results provide insight into the fluorescence enhancement mechanism. Due to the spectral overlap of the plasmonic profile with the absorption spectrum of the dye, an enhanced excitation rate may be reached. Thus the fluorescence intensity is enhanced because the fluorophore is excited more often [32]. This mechanism may be the explanation for the signal increase seen with sample 1 and 2 for fluorescence measurements of Cy3.5. A further contribution to SEF is described as an enhanced decay rate that improves the quantum yield of the fluorophore and decreases the lifetime, which should allow the fluorophore to undergo more excitation–de-excitation cycles before photobleaching occurs [26,32].

## Conclusion

In this paper, the application of a sensor platform employing fluorescence and SERS detection was introduced. Since the signal intensity is known to show a strong dependence on the distance between the molecules of interest and the metallic surface of the plasmonic active particle [27–28], an array of periodically patterned gold nanostructures mounted on a quartz wafer was preferred for these multimodal readout applications, due to the feasibility of binding molecules to the quartz layer, resulting in different distances to the metallic surface. In studying the example of a DNA detection scheme with a Cy3.5 dye label, fluorescence spectroscopy was applied, due to its fast detection time of several seconds, in order to detect the binding event of complementary DNA on the biochip surface. Additionally, SERS provides fingerprint information of the dye-label and the results illustrate the mismatch selectivity. By careful adjustment of the plasmonic behavior, the fluorescence intensity of the dye-label was significantly increased. As a result, the tuning of the optical behavior of plasmonic arrays allows studies of the fluorescence and SERS enhancement mechanism in future work. Finally, this study is a contribution towards the development of more flexible applications of the same biochip platform, through the performance of both fluorescence and SERS microscopy.

## Experimental

**Fabrication process of periodically patterned gold nanorhomb arrays.** The geometry of the plasmonic array was optimized for maximum signal enhancement by finite element method (FEM) simulations (COMSOL Multiphysics). Periodically patterned SERS arrays were fabricated by means of electron beam lithography and Argon ion beam etching. Quartz wafer was coated with 20 nm of gold by vacuum evaporation, followed by spin-coating of a 120 nm thick PMMA (polymethyl methacrylate) resist layer onto the metal film. The resist layer was exposed by a commercial e-beam writer (LION LV-1, Vistec Electron Beam GmbH) operating at 20 keV. After the development in organic solvents (60 s in MIBK:IPA = 1:1 solution), the unprotected gold layer was removed by Argon ion beam etching. Finally, the entire process was completed with oxygen plasma cleaning. The fabrication process was described in detail previously [21–23,34]. Based on the concept of the crossed exposure of two gratings of lines in the resist layer, regularly patterned gold surfaces were produced. The gold surfaces were characterized by means of SEM and optical far-field transmission measurements (Lambda-950 Perkin Elmer).

**Sample preparation (DNA immobilization).** The regularly patterned gold nanorhomb arrays were treated with oxygen plasma under gentle conditions (35 W, 30 s) before usage. To prevent nonspecific interactions of the DNA strands with the metallic surface through the DNA bases, the plasmonic arrays were incubated in a 10 mM ethanolic solution of octanethiol, providing a lipophilic protection layer (here: Self assembled monolayer (SAM)) on the gold surface [35]. For the binding of the amino-modified capture DNA, the quartz surface was modified with 3-glycidyloxypropyl trimethoxysilane (GOPS) as described elsewhere [36]. The capture DNA (complementary sequence NS150: amino-link-5'-TTT TTT CAG CAT GTG CTC CTT GAT TCT ATG-3'; one mismatch sequence NS151: amino-link-5'-TTT TTT CAG CAT GGG CTC CTT GAT TCT ATG-3'; three mismatch sequence NS153: amino-link-5'-TTT TTT CAG CAT TAT CTC CTT GAT TCT ATG-3'; negative control N7 5'-ACT GAC TGA CTG ACT GAC TGA CTG GGC GGC GAC CT-3'-amino-link) was prepared as a 1 µM solution in 5× phosphate buffered saline (PBS). To deposit small volumes (here: 4 nL) of the capture DNA solution, a Nano-Plotter NP 2.0 (GeSiM mbH, Großerkmannsdorf, Germany) was used. After the drops dried up, the complete immobilization of the capture DNA strand was ensured by an UV linking process [37] (5 min at 254 nm). Finally, the chips were thoroughly washed to remove all unbound capture DNA. Before the hybridization, the dye-labeled target DNA (50 nM Cy3.5-labeled sequence: Cy3.5-5'-CAT AGA ATC AAG GAG CAC ATG CTG AAA AAA-3') was suspended in 5× saline–sodium citrate (SSC) and 0.1% sodium dodecyl sulphate (SDS). Droplets of approximately 10 μL of the target DNA were added onto the chip and incubated for 1 h at 40 °C in a humidity chamber. Afterwards, the substrates were washed for 5 min each in 2× SSC and 0.1% SDS, 2× SSC and 0.2× SSC. Finally, the chips were dried under a stream of nitrogen.

**Fluorescence measurements.** Fluorescence images were recorded by means of an Axio Imager A1m microscope with an AxioCam MRc5 camera (Carl Zeiss Jena GmbH, Germany). The correct settings of excitation and emission wavelength for the samples were realized by the special filter set 77HE (Carl Zeiss Jena GmbH, Germany), which contains a through-hole bandpass filter with specific excitation wavelengths in the range of 483 nm, 564 nm and 642 nm. Excitation and emission spectra of the fluorescent dye Cy3.5 were measured with a Jasco FP-6200 spectrofluorometer (JASCO Germany).

**SERS set-up.** The Raman spectra were recorded with a micro-Raman setup (HR LabRam invers, Jobin-Yvon-Horiba). The spectrometer has an entrance slit of 100 µm, a focal length of 800 mm, and is equipped with a 300 line mm^−1^ grating. The 633 nm line of a He–Ne laser (Coherent) with a laser power of ~600 µW incident on the sample was used for excitation. The Raman scattered light was detected by a CCD camera operating at 220 K. A Leica PLFluoar ×100 objective (NA 0.75) was used for focusing the laser light onto the samples (focus size ~1 µm) and collecting the Raman signal.
